# Current Use of Baricitinib in COVID-19 Treatment and Its Future: An Updated Literature Review

**DOI:** 10.7759/cureus.28680

**Published:** 2022-09-01

**Authors:** Derma Dupuis, Kasinda Fritz, Emeka Ike, Oyinkansola Arogundade, Enoch O Adewara, Esther O Monday, Bolaji O Ayinde

**Affiliations:** 1 Department of Research, All Saints University, School of Medicine, Roseau, DMA; 2 Faculty of Medicine, All Saints University, School of Medicine, Roseau, DMA

**Keywords:** janus kinase inhibitors, jak-stat, coronavirus, sars-cov-2, covid-19, baricitinib

## Abstract

The levels of infectivity and mortality that ensued due to the coronavirus disease 2019 (COVID-19) pandemic caused an apparent global outcry. The health system, burdened by increasing deaths and hospitalizations, sought more effective treatment. This necessitated scientists and researchers to utilize existing drugs such as baricitinib, which has proved itself as anti-inflammatory and immunomodulatory. A qualitative systematic review was conducted using databases such as Google Scholar, Science Direct, PubMed, and BioMed Central to locate relevant articles published from 2019 onward on the effectiveness of baricitinib. After evaluation of the full-text articles, 16 were selected for review. Overall, baricitinib was seen as beneficial in decreasing respiratory failure and the use of mechanical ventilation, also preventing deterioration of COVID-19 symptoms. When used as a single agent or combined with other drugs, baricitinib improves the peripheral capillary oxygen saturation (SpO2)/fraction of inspired oxygen (FiO2) ratio. The drug does not introduce any major side effects, but a mild increase in liver enzymes has been observed. Baricitinib proves to be a safe and effective treatment for COVID-19. Administered as monotherapy or in conjunction with other drugs, baricitinib provides tremendous clinical benefit to infected patients and shows good potential in terms of efficacy for future COVID-19 regimens.

## Introduction and background

Repurposing pharmaceutical drugs for uses other than the originally developed intent is not a novel idea. Many drugs have been repurposed to treat various diseases; for instance, thalidomide, a sedative, and a well-known teratogen has been repurposed to treat multiple myeloma [1]. The coronavirus disease 2019 (COVID-19) pandemic presented a clarion call to pharmaceutical companies and health professionals to explore innovative ways to reposition already established pharmaceutical agents to treat COVID-19 and its associated secondary clinical manifestations. Hospitalized severe acute respiratory syndrome coronavirus 2 (SARS-CoV-2)-infected patients often experience hyperinflammation leading to septic shock, acute respiratory distress syndrome (ARDS), and death [2]. It is for this reason that recycling drugs such as baricitinib is necessary for rapid and effective response in patients in hyperinflammatory states. 

The severity of COVID-19 can be attributed to the pervasive way it attacks multiple organ systems in the body. One of the recognized clinical manifestations of COVID-19 infections is bilateral pneumonia associated with ARDS [3]. Internally, this ARDS triggers an excessive immune reaction called a cytokine storm [4]. According to Hu et al., this storm generates hyperinflammatory cytokine cells and chemical mediators and is indicative of the severity of the infection and probability of death from the disease. The development of oral, reversible, and selective Janus kinase (JAK) 1/JAK 2 inhibitors like baricitinib has served as a treatment for individuals with long-standing rheumatoid arthritis (RA) and has the capability to suppress systemic inflammatory responses, thereby minimizing the risk of a cytokine storm [3]. In the study by Quek et al, a justification was suggested for its usage in COVID-19 as it inhibits the JAK signal transducer and activator of transcription (JAK-STAT) signaling pathway, which is exploited by many cytokines and plays an important role in cytokine release syndrome [5]. 

Several studies in the literature were reviewed on the mechanism of action for baricitinib in treating moderate to severe cases of COVID-19. Some highlight a combined therapeutic approach and others, a mono-therapy. It should be noted that the patients in all of the studies were not already using baricitinib for RA. Over time, the efficacy and safety of baricitinib in the treatment of RA have been moderately researched and its similarity to other JAK inhibitors such as tofacitinib has been evaluated. Lexicomp, Inc. (Hudson, Ohio, United States) reported similar mechanisms of action and similar risks of both baricitinib and tofacitinib [6]. To determine its long-term safety, Taylor et al. pooled data from nine randomized clinical trials on patients with active RA [7]. Through over nine years of treatment, baricitinib maintained a constant safety profile. Given this safety record, it is expected that it will not be a problem when utilized for COVID-19 therapy.

Objective and significance of the study

This research is intended to examine the effectiveness of the current use of baricitinib in the treatment of COVID-19 and to determine if the drug has a role in the primary treatment of COVID-19. The objective is to determine the effectiveness of baricitinib in the treatment of COVID-19 and highlight possible futuristic improvements to its current use.

Occupying the position of its notoriety, the SARS-CoV-2 virus continues to pique the interest of many, globally. Traditionally, treatment options have stemmed from conventional therapy. Interestingly, there is a significant leaning towards non-conventional regimens, using drugs well known to treat other conditions. Baricitinib falls in this category, and it holds the center position in this discourse. Hence the significance of this study.

Materials and method

**Figure 1:**
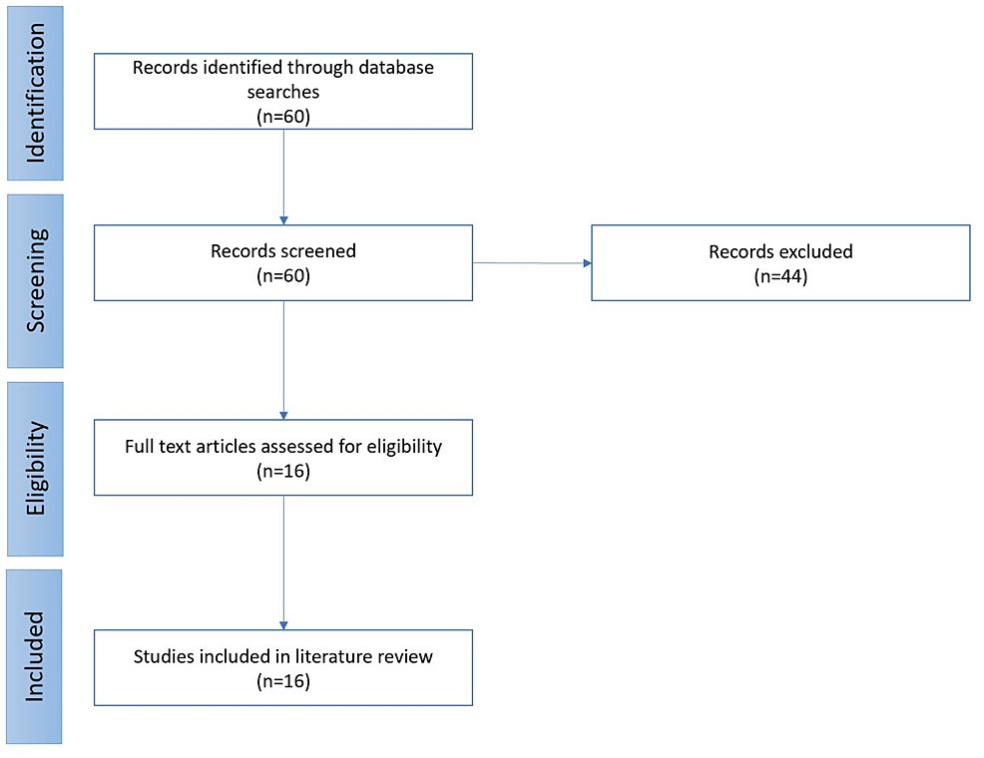
PRISMA flowchart showing article selection process for review PRISMA: Preferred Reporting Items for Systematic Reviews and Meta-Analyses

A systematic review was conducted using various search engines and index databases including Google Scholar, BioMed Central, Science Direct, and PubMed (Figure 1). The major selection criterion was articles focused on the use of baricitinib in the treatment of COVID-19 between December 2019 and May 2022. This review also included articles containing baricitinib as part of combined drug therapy for the same purpose. The arrangement of the data using the Preferred Reporting Items for Systematic Reviews and Meta-Analysis (PRISMA) flowchart provided a guide for the selection process. The abstracts of various articles followed by the full-text articles were carefully selected and reviewed. Studies that did not comprehensively cater to the discourse were excluded. Regular consultations among all authors provided tangible and meaningful criticism. "Baricitinib", "COVID-19", "SARS-CoV-2", "Coronavirus", "JAK-STAT", "Janus kinase inhibitors" were the specific keywords used while searching for articles.

Quality assessment

After article selection and review, the Scale for the Assessment of Narrative Review Articles (SANRA) was used to ascertain the quality of this review. SANRA consists of six categories based on data presentation, description, importance, and referencing of the literature. Each category has a maximum score of 2. The articles included in our study scored 11/12 based on our assessment using SANRA guidelines.

Baricitinib: mechanism of action

Baricitinib is a JAK1/2 inhibitor; an immunomodulator with anticytokine properties that stifles immunologic response making it useful for diseases associated with excessive cytokine release, like COVID-19 and RA [2,3]. It also prevented viral endocytosis by specifically targetting host proteins [2]. In the immunopathogenesis of COVID-19, when the virus infiltrates the host cells, the immune system recognizes this invasion and signals the pathway for cytokine release, which attracts B and T cells. Ordinarily, immunoactivity is reduced when the lymphocytes clear the virus but this is not the case in COVID-19, the pro-inflammatory cytokines lead to an exaggerated response which causes tissue damage and eventually leads to diffuse alveolar damage, multi-organ failure, and septic shock [5]. The role of baricitinib in the treatment of COVID-19 had to do with its inhibition of the JAK-STAT signaling pathway which is utilized by many cytokines for their activation and release, it also inhibits a protein that controls viral endocytosis called AP2-associated protein kinase [2,5].

As effective as baricitinib is, it should only be used in COVID-19 patients with significant oxygen requirements or those with prominent systemic inflammation and in patients with RA that have not achieved expected treatment goals despite optimal therapy with methotrexate. It should be avoided in patients with an absolute lymphocyte count of <200 cells/mm3 or an absolute neutrophil count of <500 cells/mm3 and in combination with strong immunosuppressants or biologic disease-modifying antirheumatic drugs (DMARDs) [6,7].

## Review

The mortality associated with cases of critically ill COVID-19 patients complicated by various comorbidities has been staggering. Baricitinib proves to be a worthy contender in reducing mortality in these severely ill patients by down-regulating inflammatory mediators implicated by COVID-19 pathophysiology two days after the start of treatment and reducing cytokine and chemokine production [2-4]. A randomized placebo-controlled trial conducted by Ely et al. shows that mortality is decreased upon treatment with baricitinib in patients without any spiking rates of infections, clots, or cardiovascular events [2]. Maslova et al. reported that even though liver enzymes such as transaminases were slightly elevated in some patients using baricitinib, the use of the drug resulted in both a drop in the frequency of death and the requirement of invasive lung ventilation [3]. Moreover, baricitinib showed a similar safety profile to that of standard care, which was echoed by Rosas et al. reporting no severe side effects [8]. According to Rosas et al., baricitinib was administered to a group of patients presenting with a worse partial pressure of arterial oxygen (Pa02) and fraction of inspired oxygen (Fi02), and no one in the group was admitted into the ICU [8]. This is particularly intriguing as the patients had concomitant chronic diseases.

It was observed that the clotting issue noticeable in some COVID-19 treatments was absent with this drug. Orantes et al. noted improvements in patients with severe COVID-19 pneumonia when treated with baricitinib [9]. Similarly, Stebbing et al. noted that baricitinib produced good outcomes even with the concomitant use of steroids [10]. A similar outcome was observed by Melikhov et al. [11]. Marconi et al. observed a 5% difference in mortality reduction between patients using baricitinib as compared to those on placebo [12]. Likewise, Abizanda et al. recorded that treatment with baricitinib resulted in a 54% and 48% reduction in mortality from any cause in patients under the age of 70 and patients over 70 years, respectively [13]. This therapy was linked with an 8.1% reduction in 30-day absolute mortality risk in patients below 70 years, and an 18.5% reduction in 30-day absolute mortality risk in those aged 70 and above. 

As adjunctive therapy, Rodriguez-Garcia revealed that a baricitinib-corticosteroid combination was associated with higher rates of improvements in lung function as well as the betterment of SpO2/FiO2 ratio in patients suffering from moderate to severe COVID-19 pneumonia, indicating a synergistic effect on increasing pulmonary function when combined with corticosteroid therapy [14]. Furthermore, observations from Cantini et al. showed that all clinical, analytical, and pulmonary indicators improved markedly, except for ageusia/anosmia, as well as evidence of significantly reduced CRP and IL-6 levels. The data reported bolstering the efficacy and safety of baricitinib in patients with moderate COVID-19 pneumonia [15]. In addition, this observation is supported by Pérez-Alba et al. whose study showed that giving baricitinib with dexamethasone, a corticosteroid, markedly decreased the 30-day mortality rate compared to dexamethasone monotherapy [16]. Supporting evidence points to the use of baricitinib in conjunction with other drugs. The use of baricitinib as combined therapy with remdesivir proved to be quite effective compared to monotherapy of the alternate drug (remdesivir). Some studies using combined therapy with hydroxychloroquine stated that the survival rate of COVID-19 patients with moderate to severe symptoms was 80%. Although these groups had small sample sizes and registered a 20% mortality, these were mainly patients suffering from underlying conditions [17,18].

Hasan et al. organized a study with 238 hospitalized patients in Bangladesh, which supported the notion that high-dose baricitinib reduces the number of recovery days, decreases the need for ICU support owing to the aggravated symptoms, and leads to early recovery time [19]. A follow-up similar study comprising 37 patients cements further the notion that this drug will produce positive results within 14 days, provided treatment begins within a short time from admission. In Table 1, the general characteristics and findings of various trials and studies on the use of baricitinib in COVID-19 were collated and summarized.

**Table 1:** Summary of baricitinib use in COVID-19 in different studies COVID-19: coronavirus disease 2019; SpO2: oxygen saturation; FiO2: fraction of inspired oxygen; SARS-CoV-2: severe acute respiratory syndrome coronavirus 2,  PaO2: partial pressure of arterial oxygen; CRP: C-reactive protein; IL-6: interleukin 6; IMV: interim monitoring visit; ECMO: extracorporeal membrane oxygenation; ESR: erythrocyte sedimentation rate

Author	Design features	Sample Size	Main findings
Maslova et al. [3]	Two groups of 20 individuals were divided into an experimental and control group	40	Respiratory failure was reduced as well as the use of oxygen masks in groups that were on baricitinib in contrast to the group that was not taking the drug.
Rosas et al. [8]	Retrospective observational study	60	Treatment with baricitinib did not introduce any serious side effects and patients that took the drug as monotherapy did not require admission to the Intensive care unit and a smaller number of individuals died compared to the other study groups.
Orantes et al. [9]	Observational study	30	Patients with severe COVID-19 pneumonia receiving baricitinib therapy showed improved clinical outcomes and avoided mechanical ventilation.
Stebbing et al. [10]	Cohort studies	166	Baricitinib therapy is associated with a decrease in the deterioration of severe COVID-19 symptoms which lead to death or invasive lung ventilation.
Melikhov et al. [11]	Prospective observational series	522	Baricitinib is an ideal therapeutic option for COVID-19 pneumonia due to a resource-constrained and outpatient scenario.
Marconi et al. [12]	Phase-3 double-blind, randomized, placebo-controlled trial	1525	The 28-day mortality was 8% for baricitinib and 13% for placebo. One additional death was prevented for every 20 baricitinib-treated participants. The 60-day mortality was 10% for baricitinib and 15% for placebo.
Ely et al. [2]	Exploratory trial	101	A significant reduction in 60-day mortality was observed in the baricitinib group compared with the placebo group which showed 45% versus 62% respectively.
Abizanda et al. [13]	Retrospective cohort study	328	Patients who received baricitinib that fit in the range of age 70 and above had a reduced mortality rate than those who did not get baricitinib, and comparable results were reported in those below 70 years. No major negative effects that might be directly attributed to baricitinib were identified.
Rodriguez-Garcia et al. [14]	Prospective observational study	112	Compared to the corticosteroids-only group, the baricitinib-corticosteroids group showed a larger improvement in SpO_2_/FiO_2 _ratio from hospitalization to discharge. Baricitinib alongside corticosteroids was linked with higher improvement in pulmonary function in patients with moderate to severe SARS-CoV-2 pneumonia.
Cantini et al. [15]	Retrospective multicenter study	191	Except for ageusia/anosmia, all clinical, analytical, and respiratory parameters improved considerably. When compared to baseline data, SpO_2_ substantially improved at week two and PaO_2_/FiO_2_ markedly improved in the first two weeks. The baricitinib group had significantly reduced CRP and IL-6 levels.
Pérez-Alba et al. [16]	Retrospective comparative study	197	In patients with severe COVID-19, the addition of baricitinib to dexamethasone decreased mortality, but no difference was noted in the incidence of IMV
Kalil et al. [17]	Double-blind, randomized, placebo-controlled trial	1033	Patients had a shorter recovery time when baricitinib combined with remdesivir compared to remdesivir and placebo. ECMO or high-flow oxygen with the combined therapy was even more effective.
Titanji et al. [18]	Retrospective cohort study	15	Normalization of body temperature and fall in ESR, IL-6, CRP, and cytokines are most notable.
Hassan et al. [19]	Prospective cohort study	238/37	Baricitinib was given in 8 mg and 4 mg amounts to form high dose and low dose groups respectively.
Stebbing et al. [20]	Case series	4	Use artificial intelligence to formulate predictions and so treat patients thus reducing admission to the Intensive care unit and mortality
Bronte et al. [21]	Longitudinal Trial	20	Marked reduction in interleukins and need for oxygen support

The future application of the use of baricitinib in COVID-19 management seems exciting. According to Stebbing et al., the use of algorithms can be used to match the efficacy of other drugs used to treat COVID-19 [20]. Artificial intelligence using baricitinib mechanism, that is, anti-cytokine, anti-inflammatory, antiviral functions (numb associated kinase) among others can be further developed. The goal is to significantly reduce the effect of this current public health issue.

Bronte et al. underscore the agreed mechanism of baricitinib functioning. Patients recorded a reduction in serum cytokine levels especially of IL-6, IL-1B, and tumor necrosis factor (TNF)-a, increasing frequencies of T- and B-cells, and increased antibody against spike proteins with reduced need for oxygen support [21]. Although, Ejaz et al. report that comorbid individuals must adopt vigilant preventive measures and scrupulous management [22]. Having understood the underlying mechanism, the idea is to capitalize and medically advance. 

Study limitations

Research on the novel COVID-19 virus and treatment is still ongoing. This review was conducted during the winter semester and the study took about three months, and this could have easily influenced a selection bias. Furthermore, having little time meant that the number of articles assessed, including those that compared the efficacy and potency of baricitinib with other COVID-19 drugs, may have been inadequate. Within such constraints, however, the process of information gathering and selection mitigated this drawback, and the objective was reached.

## Conclusions

Baricitinib can be administered as a monotherapy or in conjunction with other treatments with the intention of lowering the mortality rate in cases of COVID-19 infections which range from moderate to severe. Baricitinib has seen to produce remarkable clinical improvements in the participants and there is a reduced dependence on artificial ventilation by patients. Although it does not induce major adverse effects, a common observation was elevated liver enzymes. Notwithstanding, baricitinib proves to be a safe and efficacious treatment and can potentially become pivotal in the treatment of COVID-19. 

When compared to other drug therapy for COVID-19, this JAK1/2 inhibitor has no clotting adverse drug effect, which contributes to its safety profile. As for the futuristic potential of baricitinib, evidence from artificial intelligence algorithms shows it can be utilized in the reduction of the public health issues associated with COVID-19 due to its "triple threat" (anti-cytokine, anti-inflammatory, and anti-viral) function.
